# Clinical outcomes, complications and fusion rates in endoscopic assisted intraforaminal lumbar interbody fusion (iLIF) versus minimally invasive transforaminal lumbar interbody fusion (MI-TLIF): systematic review and meta-analysis

**DOI:** 10.1038/s41598-022-05988-0

**Published:** 2022-02-08

**Authors:** José Miguel Sousa, Hugo Ribeiro, João Luís Silva, Paulo Nogueira, José Guimarães Consciência

**Affiliations:** 1 Orthopaedics Department, Centro Hospitalar Lisboa Ocidental, Estrada do Forte do Alto Duque, 1449-005 Lisbon, Portugal; 2 Comprehensive Health Research Center, NOVA Medical School - Universidade NOVA de Lisboa, Campo Mártires da Pátria, 130, 1169-056 Lisbon, Portugal; 3 Área Disciplinar Autónoma de Bioestatística (Laboratório de Biomatemática), Faculdade de Medicina, Instituto de Medicina Preventiva e Saúde Pública, Universidade de Lisboa, Avenida Professor Egas Moniz, 1649-028 Lisbon, Portugal

**Keywords:** Outcomes research, Surgery

## Abstract

This meta-analysis aims to determine the clinical outcomes, complications, and fusion rates in endoscopic assisted intra-foraminal lumbar interbody fusion (iLIF) and minimally invasive transforaminal lumbar interbody fusion (MI-TLIF) for lumbar degenerative diseases. The MEDLINE, Embase, and Cochrane Library databases were searched. The inclusion criteria were: five or more consecutive patients who underwent iLIF or MI-TLIF for lumbar degenerative diseases; description of the surgical technique; clinical outcome measures, complications and imaging assessment; minimum follow-up of 12 months. Surgical time, blood loss, and length of hospital stay were extracted. Mean outcome improvements were pooled and compared with minimal clinically important differences (MCID). Pooled and direct meta-analysis were evaluated. We identified 42 eligible studies. The iLIF group had significantly lower mean intra-operative blood loss, unstandardized mean difference (UMD) 110.61 mL (95%CI 70.43; 150.80; *p* value < 0.0001), and significantly decreased length of hospital stay (UMD 2.36; 95%CI 1.77; 2.94; *p* value < 0.0001). Visual analogue scale (VAS) back, VAS leg and Oswestry disability index (ODI) baseline to last follow-up mean improvements were statistically significant (*p* value < 0.0001), and clinically important for both groups (MCID VAS back > 1.16; MCID VAS leg > 1.36; MCID > 12.40). There was no significant difference in complication nor fusion rates between both cohorts. Interbody fusion using either iLIF or MI-TLIF leads to significant and clinically important improvements in clinical outcomes for lumbar degenerative diseases. Both procedures provide high rates of fusion at 12 months or later, without significant difference in complication rates. iLIF is associated with significantly less intraoperative blood loss and length of hospital stay.

Study registration: PROSPERO international prospective register of systematic reviews: Registration No. CRD42020180980, accessible at https://www.crd.york.ac.uk/prospero/ April 2020.

## Introduction

Transforaminal lumbar interbody fusion (TLIF) has gained wide popularity among the surgical spine community due to its efficacy, safety, and reproducibility, namely in the treatment of lumbar degenerative diseases that failed conservative treatment. Several published studies favor minimally invasive TLIF (MI-TLIF) regarding intraoperative blood loss, length of stay, complication rates, and clinical outcomes over open TLIF (O-TLIF), despite higher radiation exposure^1,2^.

Minimally invasive spine surgeries have been developed to reduce tissue trauma, decrease complication rates, and improve functional recovery^3–8^. The advances in endoscopic spine surgery made way for new opportunities to minimize tissue aggression further. Recently published meta-analyses regarding the treatment of lumbar disc herniations favored endoscopic discectomy (ED) over microdiscectomy (MD) in clinical outcomes (Oswestry disability index), duration of surgery, length of hospital stay, and lower risk of overall complications. These results opened the perspective that ED could take over the place of MD as the gold standard of care in the management of lumbar disc disease^7,8^. Technological innovations in endoscopic spine surgery have widened its range of applications beyond lumbar disc herniations. Endoscopic treatment of central and lateral recess stenosis, as well as endoscopic assisted lumbar interbody fusion (LIF) for degenerative lumbar diseases, are increasingly common^9,10^.

The proposed benefits of an even less invasive technique than MI-TLIF would be further improvement in the advantages over O-TLIF and obviating general anesthesia. Encouraged by the success of ED, endoscopic assisted intraforaminal LIF (iLIF) has been increasing its popularity. Even though several surgical techniques have been described, Kambin’s triangle approach through an intraforaminal facet sparing technique is the most usual and the one that has greater potential to reduce iatrogenic soft and bone tissue trauma^10–15^. Recent studies have shown promising results regarding reduced blood loss, decreased length of stay, clinical outcomes, complications and fusion^10,14^. However, the comparison between MI-TLIF and iLIF is sparse in the literature, and concerns about the safety and effective benefits of the endoscopic technique remain unanswered.

This systematic review and meta-analysis of MI-TLIF and iLIF were conducted to synthetize and compare the available data in the literature on clinical outcomes, complications and fusion rates.

## Methods

### Literature research

This review and meta-analysis were performed following the preferred reporting items for systematic reviews and meta-analyses (PRISMA) guidelines^16^. The study protocol was registered in April 2020 with the PROSPERO international prospective register of systematic reviews (Registration No. CRD42020180980, accessible at https://www.crd.york.ac.uk/prospero/).

Electronic systematic research of MEDLINE, Embase, and Cochrane Library databases was performed to identify all relevant studies published from the date of inception to June 15, 2020. The following search strategy was used: (((“spine fusion” OR “lumbar fusion”) AND (“Endoscopy” OR “Endoscopic”)) OR ((“MI” OR “Minimally invasive”) AND (“TLIF” OR “transforaminal lumbar interbody fusion”))). The language of the included studies was restricted to English.

### Selection criteria and data extraction

For this review, “iLIF” procedures were defined as endoscopic assisted lumbar interbody fusion performed through a uniportal intraforaminal access to Kambin’s triangle^11–13,17^. Intraforaminal access implied that minimal, partial, or total resection of the superior articular process (SAP) was performed to allow disc space preparation and cage deployment, while the inferior articular process (IAP) had to be preserved. Studies reporting percutaneous LIF or endoscopic assisted transforaminal lumbar interbody fusion (TLIF) approaches with complete facetectomy were excluded. “MI-TLIF” procedures were defined as surgery performed through a muscle-sparing surgical corridor, created by serial dilators that allowed for a tubular or cylindrical retractor to be docked on the facet joint complex, as reported by Foley and Schwender^18,19^. Besides anterior support, both techniques implied supplementary same level screw fixation.

The following inclusion criteria were used: 5 or more consecutive patients who underwent iLIF or MI-TLIF for lumbar degenerative diseases; description of the surgical technique; clinical outcome measures reported at a minimum follow-up of 12 months; complications assessment; and imaging assessment of fusion at a minimum follow-up of 12 months. The corresponding authors of studies with insufficient data (i.e., reported mean, standard deviation (SD), number of subjects or events) to extract and pool the predefined primary endpoints were contacted via email for clarification, if otherwise the studies were excluded. Systematic reviews, meta-analyses, technical notes, surgical techniques, biomechanical studies, case reports, and editorials were also excluded.

Two review authors (J.M.S. and H.R.) independently retrieved and screened all titles and abstracts to determine study eligibility. Full-text articles of the relevant abstracts were reviewed by the same two authors. The reference lists of the eligible studies were hand searched for potentially relevant publications. When studies with overlapping samples and outcomes were identified, only the most complete reports included for analysis. Data extraction of the selected studies was performed independently by two review authors (J.M.S. and H.R.) using a standardized data extraction Microsoft Excel form (Microsoft, Redmond, WA). Study characteristics (number of patients, age, body mass index, disease, follow-up, number of levels operated) and outcomes were extracted. The number of subjects, mean, and SD of continuous variables, and cross tabulated frequencies of dichotomous outcomes were also extracted. The following outcomes of interest were defined: a) primary outcomes: clinical outcomes measures at baseline and last follow-up; overall complications; fusion rate; b) secondary outcomes: average surgical time, intraoperative blood loss and hospital length of stay. Clinical outcomes measures reported in at least three studies of each surgical technique were pooled for meta-analysis. Any disagreements related to study selection or data extraction were settled through discussion and consensus with a third reviewer (J.G.C.).

### Risk-of-bias

Two review authors (J.M.S. and H.R.) independently assessed the methodologic quality of the studies according to the methodological index for non-randomized studies (MINORS)^20^. Items were scored as 0, 1, or 2, whether they were not reported, reported but inadequate or reported and adequate, respectively. For non-comparative studies eight items were evaluated (maximum score of 16), and for comparative studies all 12 items were scored (maximum score of 24). Non-comparative studies with MINORS score $\le$ 12 and comparative studies with MINORS score $\le$ 20 were considered at high risk of bias. Any disagreements were resolved through discussion and consensus with a third reviewer (J.G.C.).

### Statistical analysis

Intraclass correlation coefficient (ICC) was calculated to quantify inter-rater reliability of the MINORS scores^20,21^.

Unstandardized mean differences (UMD) were pooled and calculated for continuous outcomes—Visual analogue scale (VAS) back, VAS leg, ODI, surgical time, intraoperative blood loss, hospital length of stay—as follows:$UMD\left(di\right)={\overline{x}}_{1i}-{\overline{x}}_{2i}, var\left(di\right)=\frac{{sd}_{1i}^{2}}{{n}_{1i}}+\frac{{sd}_{2i}^{2}}{{n}_{2i}}, {w}_{i}= \frac{1}{var({d}_{i})}$, where ${w}_{i}$ is the weighting factor, $di$ is the unstandardized difference of means, ${n}_{1i}$ and ${n}_{2i}$ are number of subjects in groups 1 and 2, ${n}_{i}$ is ${n}_{1i}$ + ${n}_{2i}$, ${sd}_{i}$ is the pooled standard deviation, $var\left(di\right)$ is the variance of difference, and subscript $i$ corresponds to study $i$_._

Pooled prevalence of the dichotomous outcomes was calculated as: $\overline{p}=\frac{\sum {w}_{i}{p}_{i}}{\sum {w}_{i}}$, ${w}_{i}= \frac{1}{var({p}_{i})}$ , where $\overline{p}$ was the pooled prevalence, ${p}_{i}$ was the prevalence of the event in each study, and ${w}_{i}$ was the weight of each study^22,23^.

Heterogeneity was estimated using *Q* statistics. *I*^2^ statistics was used to estimate inconsistency among the studies’ results due to heterogeneity rather than chance. Values greater than 50% were considered as substantial heterogeneity. If heterogeneity was present, between studies variation was estimated by calculating $\tau^{2}$, which was then used to calculate ${w}_{i}^{*}=\frac{1}{var\left({p}_{i}\right)+\tau 2}$, a weight term that accounted for variations between studies. Sub-group analysis was performed to explore causes of heterogeneity^24,25^.

Baseline to last follow-up mean differences of outcome measures reported in at least three studies in each group were calculated and compared to the minimal clinically important difference (MCID) of each outcome: 1.16 for VAS back, 1.36 for VAS leg, and 12.40 for ODI^26^.

UMD and odds ratio (OR) were estimated using a random effects model, with a 95% confidence interval. Statistical significance was set at *p* value > 0.05. Meta-analysis was performed between the pooled studies and the comparative study. Pooled analyses were performed using Microsoft Excel (Microsoft, Redmond, WA), the remaining statistical analyses and forest plots were performed using Cochrane Review Manager, version 5.4 (The Cochrane Collaboration, Copenhagen, Denmark)^27^.

## Results

**Figure 1:**
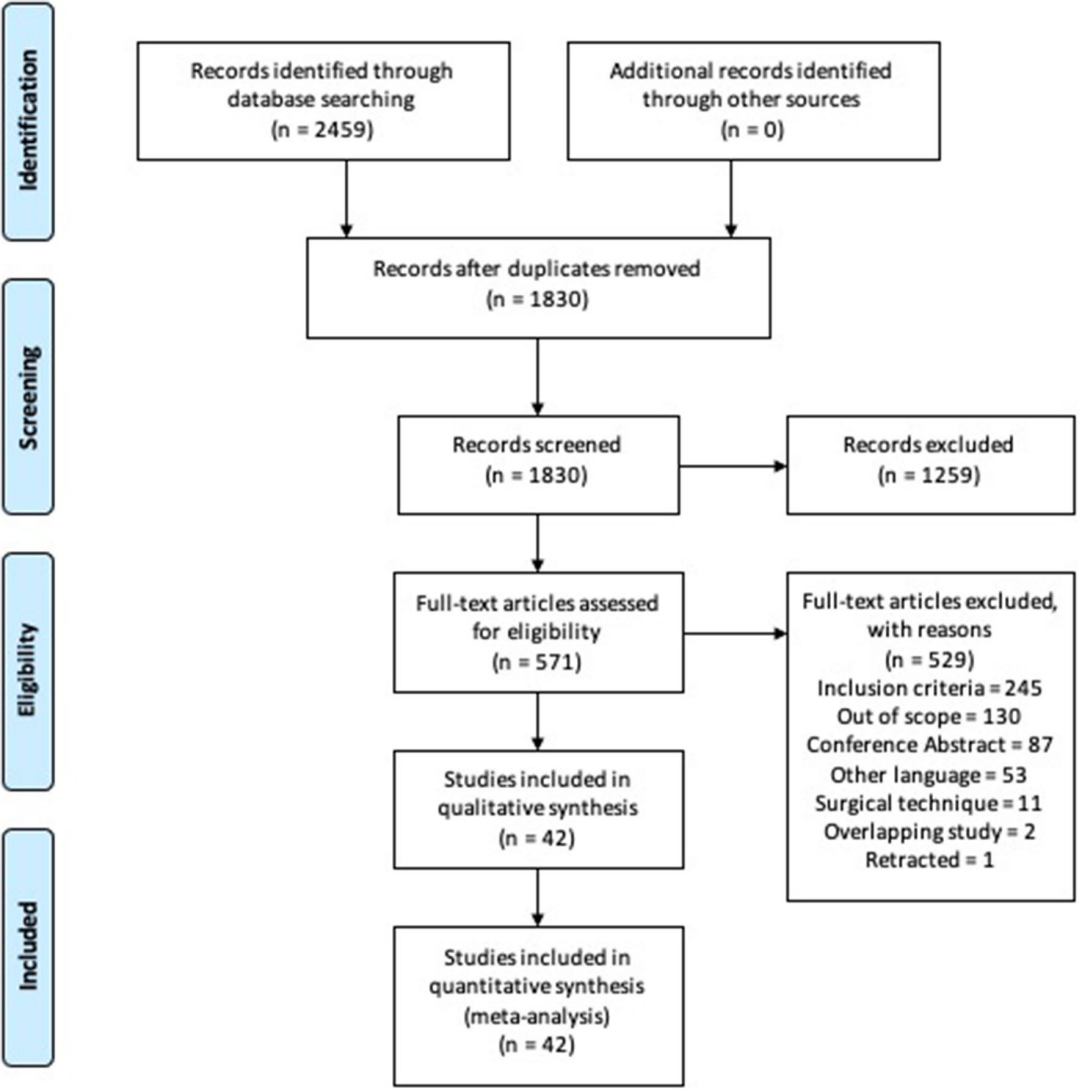
PRISMA flowchart of systematic review and meta-analysis comparing clinical outcomes and complications in endoscopic assisted intraforaminal lumbar interbody fusion (iLIF) *versus* minimally invasive transforaminal lumbar interbody fusion (MI-TLIF).

The literature search is illustrated in the PRISMA flow diagram (Fig. 1). Forty-two studies remained for qualitative and quantitative analyses^14,15,28–67^. The mean MINORS score was 12.7 $\pm$ 1.29 (10–14) for non-comparative studies, and 20.9 $\pm$ 1.9 (17–24) for comparative studies. Six non-comparative studies (MINORS score $\le$ 12) and 13 comparative studies (MINORS score $\le$ 20) were assessed as a high risk of bias. The intra-class correlation coefficient (ICC) to quantify the degree of agreement between the raters was 0.997.

The corresponding author of the study by Shen^14^ was contacted by email and provided study’s mean and SD of operative time, estimated blood loss and length of hospital stay.

### Baseline characteristics

The characteristics of the studies are summarized in Table 1. Thirty-six studies, with a total of 2076 patients, were included in the MI-TLIF group; five studies, with a total of 170 patients, were included in the iLIF group; and one prospective cohort study (PCS) with 75 patients comparing MI-TLIF to iLIF was identified.

**Table 1:** Characteristics of included studies.

Study (Level of evidence)	Journal (Year)	Years of study enrollment	MINORS score	Approach	Disease	No. of patients	Male (%)
Park et al. (IV)	Asian Spine J (2011)	–	13	MI-TLIF	DSP, DST	66	30
Rouben et al. (IV)	J Spinal Disord Tech (2011)	2002–2006	13	MI-TLIF	DDD, DSP, DST	169	43
Lee et al. (II)	Eur Spine J (2012)	2002–2008	22	MI-TLIF	DSP, DDD, DST	72	28
Lee et al. (IV)	Spine (2012)	–	14	MI-TLIF	DST, DSP, DDD	86	52
Saetia et al. (III)	J Med Assoc Thai (2013)	2008–2009	13	MI-TLIF	DSP	12	8
Seng et al. (III)	Spine (2013)	2004–2007	22	MI-TLIF	DDD, DSP, DST	40	17
Gu et al. (II)	Int Orthop (2014)	2010–2011	21	MI-TLIF	DDD, DST	44	43
Lee et al. (II)	J Spinal Disord Tech (2014)	2005–2009	14	MI-TLIF	DDD, DSP, DST	90	29
Min et al. (III)	Asian Spine J (2014)	2006–2011	18	MI-TLIF	DDD, DSP, DST	30	37
Shen et al. (III)	J Clin Neurosci (2014)	2009–2011	19	MI-TLIF	DDD, DST	34	47
Adogwa et al. (III)	World Neurosurg (2015)	2003–2010	20	MI-TLIF	DDD, DSP, DST	40	50
Brodano et al. (III)	J Spinal Disord Tech (2015)	2006–2010	21	MI-TLIF	DDD, DSP	30	60
Kuo et al. (III)	Neurosurg focus (2016)	2007–2012	22	MI-TLIF	DSP	22	27
Li et al. (II)	J Spinal Disord Tech (2015)	2008–2009	22	MI-TLIF	DDD, DSP, DST	95	48
Yang et al. (II)	Int J Clin Exp Med (2015)	–	24	MI-TLIF	DDD, DSP, DST	50	36
Fan et al. (III)	J Clin Neurosci (2016)	2010–2014	22	MI-TLIF	DSP	78	60
Gao et al. (III)	Biomed Res. (2016)	2011–2014	19	MI-TLIF	DDD, DSP	75	40
Kim et al. (IV)	World Neurosurg (2016)	2011–2013	14	MI-TLIF	DDD, DSP	50	44
Shen et al. (II)	Clin Spine Surg (2016)	2009–2011	20	MI-TLIF	DDD	34	47
Lv et al. (III)	Minim Invasive Ther Allied Technol (2017)	2010–2012	20	MI-TLIF	DDD	50	–
Razak et al. (IV)	Asian Spine J (2017)	2004–2009	14	MI-TLIF	DSP	56	29
Serban et al. (II)	Biomed Res Int (2017)	2011–2015	19	MI-TLIF	DSP, ISP	40	40
Yang et al. (IV)	Int J Clin Exp Med (2017)	2010–2014	14	MI-TLIF	DSP, DST	65	51
Yang et al. (II)	J Orthop Traumatol (2017)	–	21	MI-TLIF	DSP, DST	21	33
Zhang et al. (III)	Medicine (2017)	2012–2014	19	MI-TLIF	DSP, ISP	26	42
Wu et al. (III)	Ann Transl Med (2018)	2010–2015	17	MI-TLIF	DSP, ISP	79	42
Wu et al. (IV)	Biomed Res Int (2018)	–	11	iLIF	DSP, ISP	6	50
Zhao et al. (III)	Int Orthop. (2018)	2014–2015	20	MI-TLIF	DST, DSP, ISP	129	50
Goh et al. (III)	Clin Orthop Relat Res (2019)	2012–2014	20	MI-TLIF	DSP	78	24
Kolcun et al. (IV)	Neurosurg Focus (2019)	2014–2017	13	iLIF	DDD, DSP	100	44
Lin et al. (III)	Neurol Med Chit (Tokyo) (2019)	2010–2013	20	MI-TLIF	DDD	34	26
Mokawem et al. (III)	J Clin Neurosci (2019)	2015–2017	20	MI-TLIF	DDD, DSP, DST, DSS	50	–
Shen (IV)	World Neurosurg (2019)	–	12	iLIF	DSP, DST	18	–
Yang et al. (IV)	Biomed Res Int (2019)	2016–2017	12	iLIF	DST	7	14
Zhao et al. (IV)	J. Orthop. Surg. Res. (2018)	2008–2014	12	MI-TLIF	DSS	22	36
Zhao et al. (III)	Eur J Inflamm (2019)	2014–2015	19	MI-TLIF	DDD, DST	45	–
Ao et al. (II)	Int J Surg. (2020)	2018	22	Both	DSP, DST	75	51
Chan et al. (III)	Neurosurgery. (2020)	2014–2016	22	MI-TLIF	DSP	72	44
El Mansy et al. (III)	Musculoskelet Surg. (2020)	2011–2014	22	MI-TLIF	DDD, DST	15	60
Jin et al. (IV)	Pain Physician (2020)	2016–2017	10	iLIF	DDD, DST	39	64
Kim et al. (III)	Clin. Spine Surg. (2020)	2015–2018	21	MI-TLIF	DSP, ISP	55	45
Wang et al. (IV)	J Int Med Res (2020)	2016–2017	11	MI-TLIF	DSP, DST	122	37

Study (Level of evidence)	Female (%)	BMI, Mean ± SD, (Range)	Clinical follow-up, Mean ± SD, (Range). mo	Age, Mean ± SD, (Range), yr	Single-level	> 2 levels	Outcome measures
Park et al. (IV)	70	–	36.1 ± 9.9 (24–63)	57.5 ± 9.2	66	–	VAS back, VAS leg, ODI
Rouben et al. (IV)	57	29.7 ± 6.8 (18.3–62.0)	(36–60)	44.5 ± 10.9 (17–73)	124	45	VAS, ODI
Lee et al. (II)	72	25.7 ± 4.5	24	52.2 ± 13.8	72	–	VAS back, VAS leg, ODI, SF-36, NASS
Lee et al. (IV)	48	–	25	57.6 ± 13.3	73	13	VAS back, VAS leg, ODI
Saetia et al. (III)	92	25.14 ± 3.69 (17.58–30.70)	28.1 (24–38)	63.1 ± 6.84 (54–73)	12	–	VAS back, VAS leg, ODI
Seng et al. (III)	83	25.3 ± 0.67	60	56.6 ± 1.63	40	–	VAS back, VAS leg, ODI, SF-36, NSS
Gu et al. (II)	57	–	20.6 ± 4.5	66.4 ± 6.7	–	44	VAS back, VAS leg, ODI
Lee et al. (II)	71	25.3 ± 4.3	24	52.2 ± 14.1	90	–	VAS back, VAS leg, ODI, SF-36, NSS
Min et al. (III)	63	22.81 (17.7–29.7)	24.53 (12–52)	56.1 (30–75)	30	–	VAS leg, ODI
Shen et al. (III)	53	–	26.6 (18–36)	58.9 ± 10.1	34	–	VAS, ODI
Adogwa et al. (III)	50	34.48 ± 4.39	24	56.6 ± 11.7	40	–	VAS back, VAS leg, ODI, SF-36
Brodano et al. (III)	40	–	23 (12–38)	46 (28–56)	30	–	VAS back, ODI
Kuo et al. (III)	73	25.2 ± 3.0	32.5 ± 16.8	57.2 ± 11.6	22	–	VAS back, VAS leg, ODI, JOA
Li et al. (II)	52	23 ± 6.8	51.8 ± 6.8	56 ± 7.8	95	–	VAS back, VAS leg, ODI
Yang et al. (II)	64	–	24	58 ± 13.4	50	–	VAS back, VAS leg, ODI, JOA
Fan et al. (III)	40	22.27 ± 1.49	30.78 ± 14.15	60.95 ± 9.06	–	–	VAS, ODI, JOA, MacNab
Gao et al. (III)	60	–	12	53 ± 8	75	–	VAS, ODI
Kim et al. (IV)	56		18.1 ± 6.6	58.1 ± 14.6	38	12	VAS back, VAS leg, ODI
Shen et al. (II)	53	–	26.6 (18–36)	58.9 ± 10.1	34	–	VAS, ODI, mProlo
Lv et al. (III)	–	–	36	–	50	–	VAS back, VAS leg, ODI
Razak et al. (IV)	71	25.7 ± 3.7	60	53.7 ± 11.3	56	–	VAS back, VAS leg, ODI, SF-36, NSS
Serban et al. (II)	60	28.97 ± 5.18 (21–40)	12	51.3 ± 9.36 (34–69)	40	–	ODI
Yang et al. (IV)	49	23.5 ± 1.77	> 20	57.8 ± 12.8	65	–	VAS back, VAS leg, ODI, JOA, MacNab
Yang et al. (II)	67	23.7 ± 2.9	24	63.5 ± 9.1	21	–	VAS back, VAS leg, ODI
Zhang et al. (III)	58	–	28 ± 3.6 (24–32)	47.2 ± 7.7	26	–	VAS, ODI
Wu et al. (III)	58	–	> 24	58.1 ± 12.8	79	–	VAS back, VAS leg, ODI
Wu et al. (IV)	50		35.1 ± 3.0 (31.5–38.1)	56 ± 13.0 (33–72)	6		VAS back, VAS leg, ODI, SF-36
Zhao et al. (III)	50	23.17 ± 1.7	23.27 ± 9.5	61.7 ± 14.6	–	129	VAS back, VAS leg, ODI, MacNab
Goh et al. (III)	76	24.5 ± 4.5	> 24	67.5 ± 5.4	78	–	VAS back, VAS leg, ODI, SF-36, NSS
Kolcun et al. (IV)	56		> 12	66 ± 11	84	16	ODI
Lin et al. (III)	74	24.7 ± 2.5	64.8 ± 6	65.4 ± 7.6	34	–	VAS back, VAS leg, ODI
Mokawem et al. (III)	–	28.6 ± 4.74	> 12	–	37	13	VAS back, VAS leg, ODI, EQ-5D
Shen (IV)	–	–	> 12	66 (51–82)	18	–	VAS back, ODI
Yang et al. (IV)	86	–	15 ± 3.18 (12–21)	57 ± 12.1 (43–77)	7	–	VAS back, VAS leg, ODI, MacNab
Zhao et al. (IV)	64	–	24	63.7 (47–79)	22	–	VAS back, VAS leg, ODI
Zhao et al. (III)	–	–	24	–	45	–	VAS back, VAS leg, ODI
Ao et al. (II)	49	24.9 ± 3.04	14	53.27 ± 7.36	75	–	VAS back, VAS leg, ODI, MacNab
Chan et al. (III)	56	29.5 ± 5.1	24	62.1 ± 10.6	72	–	VAS back, VAS leg, EQ-5D
El Mansy et al. (III)	40	–	24	53 (35–77)	11	4	VAS back, VAS leg, ODI
Jin et al. (IV)	36	–	23.6 ± 4.9	59 ± 9.9	36	3	VAS back, VAS leg, ODI, SF-36
Kim et al. (III)	55	–	31.5 ± 7.3	67.3 ± 10.7	55	–	VAS back, VAS leg, ODI
Wang et al. (IV)	63	24.83 ± 3.17	23.95 ± 1.43	58.28 ± 9.65	122	–	VAS back, VAS leg, ODI

*DDD* degenerative disk disease, *DSP* degenerative spondylolisthesis, *ISP* isthmic spondylolisthesis, *DST* degenerative spinal stenosis, *DSC* degenerative spinal scoliosis, *VAS* Visual Analogue Scale, *ODI* Oswestry Disability Index, *NSS* Neurogenic Symptom Score, *SF-36* short form 36, *NASS* North American Spine Society scores for neurogenic symptoms, *JOA* Japanese Orthopaedic Association scores, *EQ-5D* Euro-Qol-5 dimension questionnaire.

The mean age was 57.9 ± 11.0 years and 63.4 ± 10.9 years, for MI-TLIF and iLIF, respectively. The proportion of females was 61% for MI-TLIF, and 52% for iLIF. Thirty-two studies reported single-level surgery, two studies (MI-TLIF) reported two level surgeries, while eight studies (six MI-TLIF; two iLIF) included single-level and two or more levels surgeries.

### Operations parameters

Summary changes of surgical time, blood loss and length of hospital stay are portrayed in Tables 2 and 3.

**Table 2:** Estimation of the pooled means and prevalences for surgical mean time, blood loss, hospital stay, complications, revision and fusion in iLIF studies.

Author	N	Surgical mean time		Blood loss		Hospital stay	
		Mean	SD	Mean	SD	Mean	SD
Wu et al.	6	167.5	30.9	70	24.5	1.2	0.6
Kolcun et al.	100	91. 48	27.57	66.89	71.71	1.4	1
Shen et al.	18	168	46	35	21	1.2	0.8
Yang et al.	7	285.7	15.9	117.1	86.1	4	1.07
Jin et al.	39	213.8	31.7	25	12.6	6.7	0.9
Pooled ⍬ (95% CI)		138.32 (133.70, 142.95)		56.08 (47.27, 64.89)		2.70 (2.55, 2.84)	
*I*^2^		0.996		0.9294		0.996	
N	170	170		170		170	

Author	N	Complications		Revision		Fusion	
		Yes	No	Yes	No	Yes	No
Wu et al.	6	0	6	0	6	6	0
Kolcun et al.	100	4	96	0	100	100	0
Shen et al.	18	0	18	0	18	18	0
Yang et al.	7	2	5	0	7	7	0
Jin et al.	39	2	37	2	37	39	0
Pooled ⍬ (95% CI)		0.05 (0.00, 0.25)		0.02 (0.00, 0.13)		1.00 (1.00, 1.00)	
*I*^2^		0		0		0	
N	170	8	162	2	168	170

**Table 3:** Estimation of the pooled means and prevalences for surgical mean time, blood loss, hospital stay, complications, revision and fusion in MI-TLIF studies.

Author	N	Surgical mean time		Blood loss		Hospital stay	
		Mean	SD	Mean	SD	Mean	SD
Park et al.	66						
Rouben	169	183	63	171	107	0.6	14
Lee et al.	72	166.4	52.1	50.6	161	3.2	2.9
Lee	86	209.03	32.23	371.2	279.28		
Saetia	12	340	81.49	317	195.79	8.42	3.34
Seng	40	185	8.7	127.3	45.7	3.6	0.3
Gu et al.	44	195.5	28	248.4	94.3	9.3	3.7
Lee	90	159.1	37.48	130	122.79	2.9	1.22
Min	30	156	5.99	397.5	43.02		
Shen et al.	34	143.1	22.5	106.3	53.8	6.6	2.1
Adogwa et al.	40						
Brodano et al.	30	144		230		4.1	
Kuo et al.	22	270	78	232.5	194.2		
Li et al.	95	164.8	9.2				
Yang et al.	50	178.5	17.7	183.9	24.2		
Fan et al.	78	184.87	45.01	289.74	154.18	15.99	4.11
Gao et al.	75	191	3.2	195	203		
Kim et al.	50	266.4	84.3	112.76	61.73	2.5	1.7
Shen et al.	34						
Lv et al.	50	103.2	16.9	143.1	37.4	5.4	2.8
Razak et al.	56	167	49	126	107	2.8	11
Serban et al.	40	321.92	85.57	351.25	198.87	1.92	0.52
Yang et al.	65	184.26	20.69	190.95	28.06		
Yang et al.	21	179	20.7	188.6	42.3		
Zhang et al.	26	93	25	115	37	4.3	1.3
Wu et al.	79	145.5	21.5	163.7	49.6	5.8	1.4
Zhao et al.	129	154.8	37.95	212.22	80.98	8.04	1.97
Goh et al.	78	148	34.5			9	1
Lin et al.	34	167.1	39.1	225.2	94.1	7.8	2.3
Mokawem et al.	50						
Zhao et al.	22	153.3	26.3	175	83.4	5.4	0.9
Zhao et al.	45						
Chan et al.	72	228.2	111.5	108.8	85.6	2.9	1.8
El Mansy et al.	15						
Kim et al.	55	173	47.1			9.1	2.9
Wang et al.	122	130.48	34.44	114.1	96.7	6.45	2.47
Pooled ⍬ (95% CI)		176.44 (174.28, 178.60)		184.88 (178.69, 191.07)		5.31 (5.00, 5.63)	
*I*^2^		99.3%		98.3%		99.15%	
N	2076	1796		1568		1352	

Author	N	Complications		Revision		Fusion	
		Yes	No	Yes	No	Yes	No
Park et al.	66	3	63	3	63	51	15
Rouben	169	24	145	24	145	162	7
Lee et al.	72	7	65	1	71	70	2
Lee	86	5	81	5	81	54	32
Saetia	12	4	8	1	11	11	1
Seng	40	6	34	1	39	39	1
Gu et al.	44	5	39	0	44	41	3
Lee	90	4	86	0	90	82	8
Min	30	0	30	0	30	28	2
Shen et al.	34	3	31	1	33	34	0
Adogwa et al.	40	5	35	0	40	40	0
Brodano et al.	30	1	29	0	30	30	0
Kuo et al.	22	8	14	1	21	17	5
Li et al.	95	4	91	0	95	93	2
Yang et al.	50	5	45	0	50	44	6
Fan et al.	78	5	73	0	78	68	10
Gao et al.	75	5	70	0	75	75	0
Kim et al.	50	0	50	0	50	49	1
Shen et al.	34	3	31	1	33	34	0
Lv et al.	50	2	48	0	50	48	2
Razak et al.	56	7	49	0	56	54	2
Serban et al.	40	0	40	0	40	40	0
Yang et al.	65	14	51	2	63	55	10
Yang et al.	21	2	19	0	21	18	3
Zhang et al.	26	1	25	0	26	26	0
Wu et al.	79	6	73	3	76	78	1
Zhao et al.	129	11	118	0	129	120	9
Goh et al.	78	19	59	1	77	69	9
Lin et al.	34	18	26	2	32	32	2
Mokawem et al.	50	5	45	0	50	49	1
Zhao et al.	22	3	19	1	21	22	0
Zhao et al.	45	2	43	0	45	42	3
Chan et al.	72	6	66	1	71	72	0
El Mansy et al.	15	1	14	1	14	15	0
Kim et al.	55	3	52	1	54	51	4
Wang et al.	122	2	120	0	122	120	2
Pooled ⍬ (95% CI)		0.05 (0.00, 0.12)		0.02 (0.00, 0.05)		0.96 (0.92, 1.00)	
*I*^2^		0%		0%		0%	
N	2076	199	1887	50	2026	1933	143

In the pooled studies, surgical time was significantly increased in the MI-TLIF group (UMD 38.1; 95% CI 33.01; 43.23; *p* value < 0.0001). However, in the meta-analysis no statistically significant difference was observed between both groups (Table. 4).

**Table 4:** Unstandardized mean differences surgical time, blood loss and length of stay between MI-TLIF and iLIF.

Author	MI-TLIF			iLIF			UMD	95% CI	*p* value
	N	Mean	SD	N	Mean	SD			
**(A) Surgical time**									
Ao et al.	40	103.6	17.79	35	143	24.2	− 39.4	(− 49.1, − 29.64)	< 0.0001*
Pooled study	1796	176.4	46.75	170	138.3	30.72	38.12	(33.01, 43.23)	< 0.0001*
UMD (95% CI); I^2^	− 0.51 (− 76.45, 75.43); 99%								0.99
**(B) Blood loss**									
Ao et al.	40	171.8	84.29	35	84.29	44.34	87.5	(57.53, 117.47)	< 0.0001*
Pooled study	1568	184.9	125.1	170	56.08	58.78	128.8	(118.01, 139.59)	< 0.0001*
UMD (95% CI); I^2^	110.61 (70.43, 150.80); 85%								< 0.0001*
**(C) Length of stay**									
Ao et al.	40	5.11	1.44	35	3.11	1.18	2	(1.41, 2.59)	< 0.0001*
Pooled study	1352	5.31	5.87	170	2.7	0.95	2.61	(2.27, 2.95)	< 0.0001*
UMD (95% CI); I^2^	2.36 (1.77, 2.94); 67%								< 0.0001*

Significant values are in asterisk.

**Figure 2:**
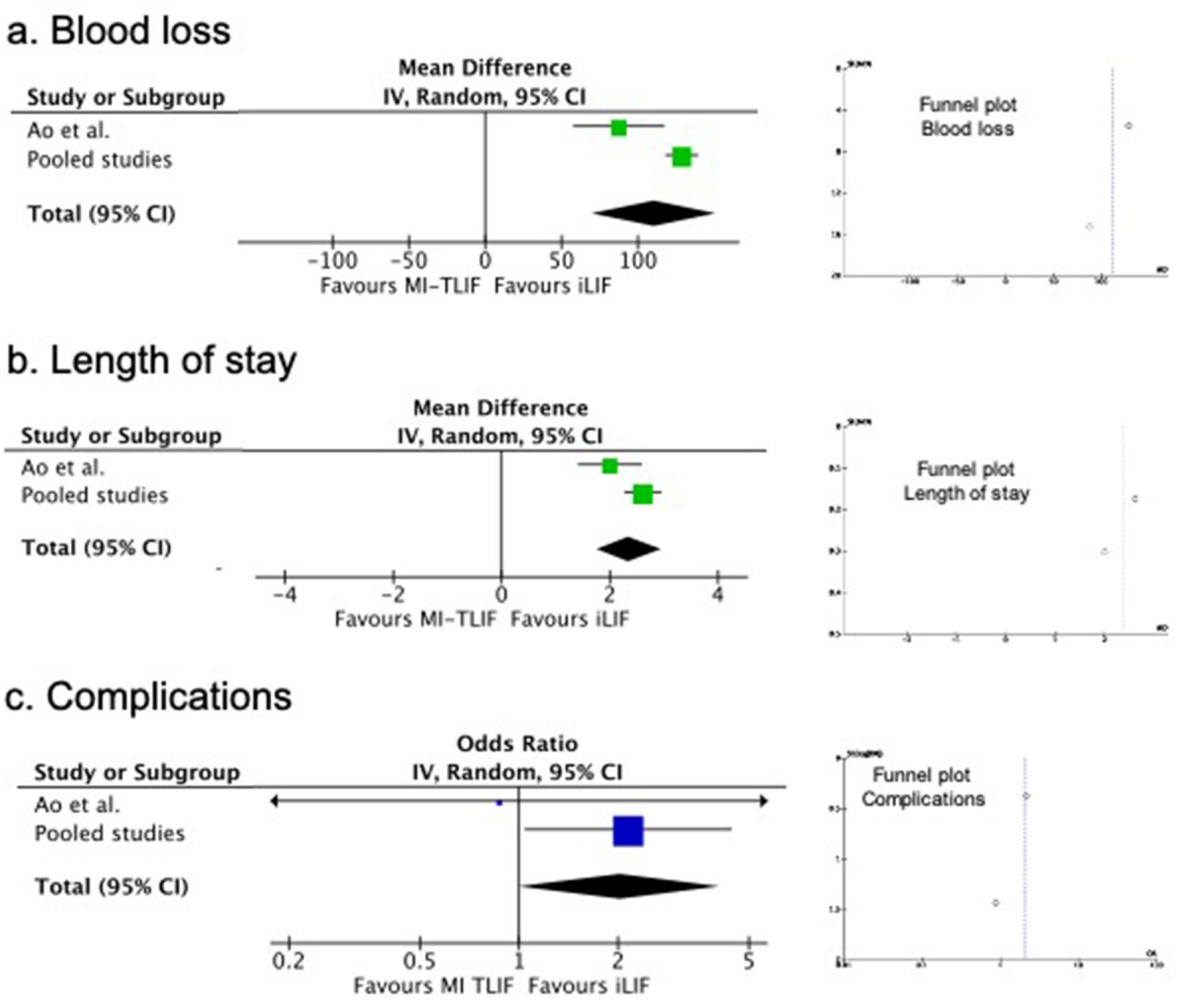
Comparison of blood loss (**a**), length of stay (**b**) and complications (**c**).

The mean intra-operative blood-loss of the pooled studies was significantly increased by 128.8 mL (95% CI 118.01; 139.59; *p* value < 0.0001) in MI-TLIF. In the meta-analysis, there was also a statistically significant mean increase of intra-operative blood-loss (UMD 110.61 mL; 95% IC 70.43; 150.80; *p* value < 0.0001) in MI-TLIF (Table. 4, Fig. 2).

In the pooled studies, the length of hospital stay had a significantly mean increase of 2.6 days (95% CI 2.27; 2.95; *p* value < 0.0001) in the MI-TLIF group. In the meta-analysis, there was also a statistically significant mean difference of 2.36 days (95% IC 1.77; 2.94; *p* value < 0.0001) favoring the iLIF group (Table. 4, Fig. 2).

### Clinical Outcomes

VAS back (33 MI-TLIF studies, n = 1946 vs 4 iLIF studies, n = 70), VAS leg (26 MI-TLIF studies, n = 1518 vs 3 iLIF studies, n = 52), and ODI (36 MI-TLIF studies, n = 2050 vs 6 iLIF studies, n = 170) were reported in three or more studies in each technique and are summarized in Tables 5 and 6. UMD of the pooled results and meta-analysis are summarized in Table 7.

**Table 5:** Estimation of the pooled means for clinical outcomes in iLIF studies.

Author	N	VAS back				VAS leg				ODI			
		Pre-op		Post-op		Pre-op		Post-op		Pre-op		Post-op	
		Mean	SD	Mean	SD	Mean	SD	Mean	SD	Mean	SD	Mean	SD
Wu et al.	6	6.17	0.75	0.67	0.52	5.33	1.97	0.17	0.41	44.83	4.75	11.17	4.31
Kolcun et al.	100	–	–	–	–	–	–	–	–	29.6	15.3	17.2	16.9
Shen et al.	18	8.1	2	1.8	0.9	–	–	–	–	48	14	13	11
Yang et al.	7	7.4	0.73	0.9	0.64	6.14	1.64	0.71	0.45	53.57	9.5	16.6	7
Jin et al.	39	5.7	0.8	0.7	0.2	7.9	0.5	1	0	43.1	4.9	16.1	7.2
Pooled ⍬ (95% CI)		6.53 (6.24, 6.81)		1.00 (0.87.1.13)		7.37 (7.1, 7.64)		0.87 (0.73, 1)		36.17 (34.22, 38.12)		16.27 (14.16, 18.37)	
I^2^		94.0%		88.8%		88.8%		90.3%		95.2%		51.5%	
N	170	70		70		52		52		170		170

**Table 6:** Estimation of the pooled means for clinical outcomes in MI-TLIF studies.

Author	N	VAS back				VAS leg				ODI			
		Pre-op		Post-op		Pre-op		Post-op		Pre-op		Post-op	
		Mean	SD	Mean	SD	Mean	SD	Mean	SD	Mean	SD	Mean	SD
Park et al	66	8.1	2	2.6	2.1	8.1	2	1.6	2.8	60.2	16.5	25.9	17.9
Rouben	169	6.9	1.45	2.67	1.96					69.3	13.5	29.2	20.5
Lee et al	72	6.3	2.9	2.3	3	5.8	3.3	1.6	2.7	48.1	18.8	21.4	20.9
Lee	86	5.5	2.68	1.78	2.08	6.8	2.35	0.62	1.2	23.7	6.25	8.81	6.85
Saetia	12	8.75	1.6	2.08	1.41					61.8	12.89	49.55	11.24
Seng	40	5.6	3.3	1.3	0.4	5.9	2.8	0.8	0.4	41.3	20.1	13.6	2.8
Gu et al	44	7.3	1.2	1.9	0.7	7.6	0.9	1.7	0.6	43.7	4.3	16.5	2
Lee	90	6.15	2.76	2.09	2.84	5.59	3.26	1.36	2.34	46.18	19.13	19.67	19.28
Min	30					7.17	0.61	0.57	0.2	23.53	2.73	7.73	1.95
Shen et al	34	7.2	2.1	2.2	1.4					51.58	16.38	22.84	15.65
Adogwa et al	40	6.97	2.49	2.42	3.81	7.07	3	3.77	4.53	50.18	16.74	11.61	25.52
Brodano et al	30	7.8	1.4	2.3	1.3					42	6.2	10	6.6
Kuo et al	22	6.9	2.1	2.9	2.6	6	2.9	2.1	2.3	23	7.7	9.9	7.1
Li et al	95	7.7	0.4	2.5	0.6	6.7	0.6	3.2	0.7	79.8	4.4	30.3	4.3
Yang et al	50	5.1	1.7	0.8	0.8	5.8	1.5	0.8	0.8	50.7	14.5	11.6	6.3
Fan et al	78	7.22	0.62	3.4	0.59					49.36	9.72	17.2	5.27
Gao et al	75	7.3	1.2	1.7	1.1					56.3	14.7	16.8	8
Kim et al	50	7.2	2.4	3.5	2.9	5.2	3.5	1.6	2.7	51	16.6	29.2	16.9
Shen et al	34	7.2	2.1	2.2	1.4					51.58	16.38	22.84	15.65
Lv et al	50	6.81	0.8	2.36	0.29	7.8	0.9	2.38	0.41	58	8.8	14.2	3.3
Razak et al	56	7	3	2	3	6	3	1	3	47	20	16	19
Serban et al	40									37.75	6.59	11.52	6.56
Yang et al	65	5.03	1.61	0.8	0.87	5.83	1.44	0.64	0.84	51.05	13.81	12.14	9.04
Yang et al	21	5.8	0.9	1	0.9	5.2	1.3	0.6	0.7	43.5	15.1	12	6.4
Zhang et al	26	7.5	1.7	1	0.6					48.6	7	11.8	2.8
Wu et al	79	6.78	1.48	1.63	1.2	7.12	1.33	1.77	1.39	60.7	10.6	25.3	6.3
Zhao et al	129	5.32	0.59	1.46	0.34	6.09	0.52	1.66	0.26	53.97	13.25	14.73	6.25
Goh et al	78	5.5	2.55	1	1.58	6	3	0	1	49	16	11.5	11.77
Lin et al	34									50.8	9.7	17.1	8.1
Mokawem et al	50	7.4	3.05	1.9	1.46	7.9	2.67	1.2	1.57	61.1	21.22	18.1	12.92
Zhao et al	22	6.2	1.8	2.2	0.7	8.2	0.7	1.4	1.4	62.4	16.1	24.2	9.3
Zhao et al	45	7.3	1.5	2	1.9	5.2	1.1	1.1	0.8	54.25	12.76	22.78	13.69
Chan et al	72	6.9	2.6	2.3	2.9	6.3	2.8	1.6	2.7	46.2	16.3	14.3	17.2
El Mansy et al	15	6.87	1.25	2.6	1.12	5.07	1.94	2.33	0.72	29.73	7.44	7.47	5.18
Kim et al	55	6.5	1.5	1.9	0.8	7.8	1.7	1.8	0.8	69.6	6.2	16.3	11.9
Wang et al	122	3.74	2.28	0.65	0.85	4.93	2.68	0.36	0.83	59.09	22.34	17.04	8.49
Pooled ⍬ (95% CI)		6.46 (6.37, 6.55)		1.96 (1.88, 2.04)		6.36 (6.24, 6.47)		1.41 (1.32, 1.50)		53.31 (52.69, 53.94)		18.54 (17.98, 19.1)	
*I*^2^		97.9%		97.7%		95.9%		98.8%		99.6%		98.3%	
N	2076	1946		1946		1518		1518		2050		2050

**Table 7:** Unstandardized mean differences and baseline to last follow-up mean differences of VAS back, leg and ODI between MI-TLIF and iLIF.

Author	MI-TLIF			iLIF			UMD	95% CI	*p* value
	N	Mean	SD	N	Mean	SD			
**(A) VAS back pre**									
Ao et al	40	5.53	1.88	35	5.09	2.09	0.44	(− 0.46, 1.34)	0.34
Pooled study	1946	6.46	1.97	70	6.53	1.22	− 0.07	(− 0.37, 0.23)	0.83
UMD (95% CI); I^2^	0.0 (− 0.34, 0.34); 9%								0.99
**(B) VAS back pos**									
Ao et al	40	1.31	1.08	35	1.18	0.95	0.13	(− 0.33, 0.59)	0.58
Pooled study	1946	1.96	1.77	70	1	0.54	0.96	(0.81, 1.11)	< 0.0001*
UMD (95% CI); I^2^	0.57 (− 0.24, 1.39); 91%								0.17
*△ VAS back									
Ao et al	40	4.22	–	35	3.91	–	–	–	–
Pooled study	1946	4.5	–	70	5.53	–	–	–	–
**(C) VAS leg pre**									
Ao et al	40	5.65	1.55	35	6.11	1.83	− 0.46	(− 1.23, 0.31)	0.24
Pooled study	1518	6.36	2.25	52	7.37	0.96	− 1.01	(− 1.29. − 0.73)	< 0.0001*
UMD (95% CI); I^2^	− 0.86 (− 1.34. − 0.37); 42%								0.0005*
**(D) VAS leg pos**									
Ao et al	40	0.79	0.86	35	0.82	0.8	− 0.03	(− 0.41, 0.35)	0.88
Pooled study	1518	1.41	1.76	52	0.87	0.49	0.54	(0.38, 0.79)	< 0.0001*
UMD (95% CI); I^2^	0.28 (− 0.27, 0.84); 87%								0.32
*△ VAS leg									
Ao et al	40	4.86	–	35	5.29	–	–	–	–
Pooled study	1518	4.95	–	52	6.5	–	–	–	–
**(E) ODI pre**									
Ao et al	40	56.9	11.5	35	53.94	10.87	2.96	(− 2.11, 8.03)	0.26
Pooled study	2050	53.31	14.5	170	36.17	13.04	17.14	(15.09, 19.19)	< 0.0001*
UMD (95% CI); I^2^	10.25 (− 3.64, 24.14); 96%								0.15
**(F) ODI pos**									
Ao et al	40	13.59	5.43	35	12.94	4.93	0.65	(-1.70, 3.00)	0.59
Pooled study	2050	18.54	12.88	170	16.27	14.08	2.27	(0.07, 4.47)	0.042*
UMD (95% CI); I^2^	1.52 (− 0.08, 3.12); 0%								0.06
*△ ODI									
Ao et al	40	43.31	–	35	41	8.43	–	–	–
Pooled study	2050	34.77	–	180	19.9	13.56	–	–	–

*△ Baseline to last follow-up mean variation.

There was no difference in baseline VAS back between pooled MI-TLIF and iLIF groups. At the last follow-up, VAS back was significantly lower in the iLIF group (UMD 0.96; 95% CI 0.81; 1.11; *p* value < 0.0001). However, the meta-analysis showed no statistically significant difference at either time-point.

VAS leg at baseline was significantly higher in the iLIF group (UMD 1.0; 95% CI 0.73; 1.29; *p* value < 0.0001), with significant heterogeneity. This statistically significant increase was also observed in the meta-analysis (UMD 0.86; 95% CI 0.37; 1.34; *p* value = 0.0005). At the last follow-up, VAS Leg was significantly lower in the pooled iLIF group (UMD 0.54; 95% CI 0.38; 0.79; *p* value = 0.027). However, the meta-analysis showed no statistically significant difference at last follow-up.

VAS back and VAS leg baseline to last follow-up mean improvement was statistically significant (*p* value < 0.0001), and clinically important (MCID > 1.16 and MCID > 1.36, respectively)^26^ for both groups.

ODI scores at baseline and last follow-up were significantly higher in the MI-TLIF group (UMD 17.1; 95% CI 15.09; 19.19; *p* value < 0.0001 and UMD 2.27; 95% CI 0.07; 4.47; *p* value = 0.042, respectively. Conversely, the meta-analysis showed no statistically significant difference at either time-point.

ODI baseline to last follow-up mean improvement was statistically significant (*p* value < 0.0001), and clinically important (MCID > 12.40) for both groups^26^.

### Complications and fusion

The overall complication rate was 4.7% for iLIF studies *versus* 9.6% for MI-TLIF. The specific complications identified in each technique are summarized in Table 6. Screw malpositioning (1.6%), adjacent segment degeneration (1.5%), and dural tears (1.3%) were the main complications in MI-TLIF, while in the iLIF group cage migration (1.1%), screw malpositioning (0.6%), and infection (0.6%) were the most prevalent complications (Table 8). The pooled OR was 2.15 (95% CI 1.04, 4.43; *p* value 0.033) when comparing MI-TLIF and iLIF. The meta-analysis showed a borderline OR (OR 2.03; 95% CI 1.01, 4.12; *p* value = 0.05) favoring iLIF (Table 9).

**Table 8:** Prevalence of complications, revision and fusion in MI-TLIF and iLIF.

	iLIF (N = 170)		MI-TLIF (N = 2076)	
Overall complications	8	4.7%	199	9.6%
Dural tear	0	0.0%	28	14.1%
Infection	1	0.6%	24	12.1%
Neurologic injury	0	0.0%	5	2.5%
Intraspinal haematoma	0	0.0%	3	1.5%
Radiculopathy	0	0.0%	6	3.0%
Dysesthesia	0	0.0%	2	1.0%
Cage subsidence	0	0.0%	14	7.0%
Cage migration	2	1.2%	11	5.5%
Screw malpositioning	1	0.6%	34	17.1%
Adjacent segment degeneration	0	0.0%	29	14.6%
Others	4	2.4%	43	21.6%
Revision	2	1.2%	50	2.4%
Fusion	170	100.0%	1933	93.1%

**Table 9:** Comparison of prevalence of complications and fusion between MI-TLIF and iLIF.

Author	MI-TLIF		iLIF		OR	95% CI	*p* value
	Yes	No	Yes	No			
**(A) Complications**							
Ao et al	1	39	1	34	0.87	(0.05, 14.48)	0.924
Pooled study	199	1877	8	162	2.15	(1.04, 4.43)	0.033
OR (95% CI); *I*^2^					2.03	(1.01, 4.09); 0%	0.05
**(B) Fusion**							
Ao et al	36	4	29	6	1.86	(0.48, 7.23)	0.364
Pooled study	1933	117	170	0	0.05	(0.00, 0.78)	< 0.001
OR (95% CI); *I*^2^					0.37	(0.01, 12.95); 81%	0.58

The pooled fusion rate had a statistically significant OR favoring iLIF (OR 0.05; 95% CI 0.00, 0.78; *p* value < 0.0001). However, the meta-analysis revealed no difference between iLIF and MI-TLIF fusion rates.

There was no significant difference (OR 2.39; 95% CI 0.58, 9.87; *p* value 0.23) in overall revision rate, 1.2% and 2.4% in iLIF and MI-TLIF, respectively (Table 9).

### Subgroup analysis

We performed subgroup analysis according to the number of levels operated. Even though ODI at baseline, and VAS back and VAS leg at last follow-up, had statistically significant differences in one level versus two level MI-TLIF, they were not clinically significant. There was no significant difference regarding complications and fusion rates.

Blood loss was significantly increased when two or more levels were operated in either technique (UMD 41.3 mL; 95% CI 27.63, 55.03; *p* value < 0.0001 for MI-TLIF; UMD 20.55 mL; 95% CI 1.5, 39.6; *p* value < 0.036 for iLIF). Both one level and two level iLIF subgroups had significantly less blood loss than one level MI-TLIF subgroup (UMD 122.02 mL; 95% CI 110.38, 133.66; *p* value < 0.0001 for one level iLIF; and UMD 101.47 mL; 95% CI 83.72, 119.22; *p* value 0.0012 for two level iLIF).

Length of hospital stay was significantly higher in the MI-TLIF subgroup of two or more levels when compared to one-level MI-TLIF (UMD 3.49 days; 95% CI 2.98; 4.0; *p* value < 0.0001). There was no significant difference between one-level subgroups of MI-TLIF and iLIF (UMD 0.33 days; 95% CI -0.008, 0.74; *p* value 0.65). Unlike surgical time and blood loss, there was no available data for length of hospital stay in two level iLIF. Comparison between one level MI-TLIF and overall iLIF length of hospital stay revealed a statistically significant decrease favoring iLIF (UMD 2.17 days; 95% CI 1.79; 2.55; *p* value < 0.0001).

## Discussion

We conducted a systematic review and meta-analysis of the available literature reporting MI-TLIF and iLIF for the treatment of lumbar degenerative diseases. We derived our conclusions based on the meta-results, given the discrepancy in some outcomes of the pooled results and the meta-analysis.

The main finding is that both MI-TLIF and iLIF provide significant clinical improvement and high rates of fusion at a minimum follow-up of 12 months. No significant difference in complication rates was identified. Furthermore, iLIF was associated with significantly less intraoperative blood loss, and reduced length of hospital stay.

Previous studies documented similar benefits of MI-TLIF over open TLIF (O-TLIF), while achieving the same fusion rates, operative time, and decreased complication rates^1,2^. On the downside, MI-TLIF was associated with increased radiation exposure. According to our findings, iLIF further enhances most of the benefits of MI-TLIF over O-TLIF. The data reported in the retrieved studies did not allow for a comparative analysis on radiation exposure.

Besides efficacy, concerns about procedure-related complications are among the major setbacks for adopting emerging surgical techniques. The present meta-analysis revealed no significant difference in complication rates. The intraforaminal route allows an anatomic approach to the disc, with total or partial preservation of the articular processes. On the one hand, facet preservation provides dural protection, reducing the risk of dural tear, on the other, direct endoscopic visualization of the nerve root allows assessing the need for further foraminal decompression, reducing the risk of nerve root damage. By providing an ultra-minimally invasive approach, trauma to the soft and bone tissues is reduced, which may account for the residual infection rates reported.

The anesthesia protocol and neuromonitoring might also play a role in the sparse number of neurologic complications reported in iLIF procedures, by allowing intra-operative neurological monitoring. Wu^15^ and Ao^62^ used general anesthesia and neuromonitoring, Shen^14^ and Kolcun^56^ operated consciously sedated patients, Jin^65^ used local anesthesia supplemented with neuroleptic analgesia for the decompression procedure, with the aim of sensory-motor separation, and epidural anesthesia when the patient complained about unbearable pain during bone harvest, cage insertion, and percutaneous pedicle screw placement procedures. Yang^59^ used a low-dose epidural anesthesia combined with local anesthesia or general anesthesia based on physical condition and willingness of patients. The vast majority of the retrieved MI-TLIF studies either did not disclosure the anesthesia protocol or used general anesthesia without neuromonitoring. Gao^44^ reported 75 patients operated under epidural anesthesia.

Studies using a strictly percutaneous approach (pTLIF) similar to iLIF have reported increased rates of post-operative radiculopathy, dysesthesia and transitory muscle weakness^68–70^. However, once endoscopic assistance was precluded, these studies were not included in our analysis. The absence of endoscopic assessment does not allow nerve root visualization or foraminal revision after cage deployment, which may justify the increased complication rates reported. From the 205 patients included in the iLIF studies, only Ao^62^ reported a patient with decreased muscular strength of quadriceps femoris, grade 4, after surgery at L4L5 level. The patient had significant relief of symptoms after one month of neurotrophic drug treatment and functional exercise.

Even though endplate preparation might be technically challenging in iLIF due to access restraints, direct endoscopic visualization of the endplates allows confirming adequate subchondral exposure^62,65^, contributing to the high rates of fusion.

In the iLIF publications, the type of cage used varied across studies. Wu^15^ and Ao^62^ used static cages, Kolcun^56^ used mesh cages, and Shen^14^, Yang^59^ and Jin^65^ used expandable cages. In most MI-TLIF studies, static cages were used or the type of cage was not disclosed, except in the study by Kim^45^ that used expandable cages. Data on sagittal alignment was insufficient, namely on iLIF studies, to derive any conclusions.

Despite overlapping and additional benefits of iLIF compared to MI-TLIF, it is worth mentioning that endoscopic spine surgeries have a steep learning curve. Knowledge and understanding of foraminal anatomy and its landmarks are of utmost importance. Besides the anesthetic technique and neuromonitoring, continuous spatial orientation of surgical instruments during the procedure is mandatory to avoid nerve root injury, either by direct trauma or excessive retraction. Pre-operative planning with magnetic resonance imaging (MRI) is crucial to assess the nerve root trajectory and eventual anatomic variations, mostly at L5S1 level where the dorsal root ganglion may be particularly endangered^10,14^. Proper training and previous experience in lumbar transforaminal endoscopic discectomy and decompression are advised.

### Strengths and limitations

Intraforaminal endoscopic assisted fusion is an emerging surgical technique. No systematic review and meta-analysis that synthesizes the available data comparing it to MI-TLIF has been published. Recently, Wagner^10^ published a review and technical note on uniportal endoscopic assisted fusion. However, despite providing a comprehensive description of the endoscopic assisted intraforaminal transkambin technique, the studies’ procedures were heterogeneous and not standardized. Our review followed the PRISMA guidelines, with a prospective design that specified the main technical details of the procedures and the outcomes and minimum follow-up to be included. Statistical analysis was performed according to previously validated statistical methodology^22–25^.

The endoscopic assisted intraforaminal transkambin approach for LIF has been termed as percutaneous endoscopic LIF (abbreviated as PELIF^15,65^ or PETLIF^59,62^) or endoscopic MIS-TLIF^56^. However, the term TLIF is misleading once endoscopic assisted intraforaminal transkambin LIF is a facet sparing procedure, as opposed to the TLIF procedure where a total facetectomy is performed. Also, the term PELIF is ambiguous once it may accommodate different approaches to perform LIF, as long as it is done percutaneously and with endoscopic assistance, either uniportal or biportal. We believe that by referring to the intra-foraminal route instead of the traditional transforaminal route, the term iLIF is more accurate and allows a comprehensive definition of the procedure.

The main limitations of our study are a consequence of the quality of the literature retrieved: low levels of evidence and heterogeneity bias. Only one prospective cohort study comparing both techniques was included, and no randomized control trials. Also, the difference in fusion definition across studies, either by evaluating X-rays or computerized tomography (CT) scans, is a source of heterogeneity and eventual bias on fusion rate assessment. The main clinical outcomes reported were pain scales (VAS back, VAS leg) and a functional/disability scale (ODI). The low prevalence of Health-Related Quality of Life (HRQoL) questionnaires prevented further evaluation and comparison of both techniques.

## Conclusion

iLIF and MI-TLIF for the treatment of lumbar degenerative diseases provide significant clinical improvement and high fusion rates at 12 months or later, without significant difference in complication rates. iLIF has significantly less intraoperative blood loss and reduced length of hospital stay in comparison to MI-TLIF.

## Supplementary Information

Supplementary Information.

## Data Availability

The datasets generated during and/or analysed during the current study are available from the corresponding author on reasonable request.
