# Supplementary material for: Clinical outcomes, complications and fusion rates in endoscopic assisted intraforaminal lumbar interbody fusion (iLIF) versus minimally invasive transforaminal lumbar interbody fusion (MI-TLIF): systematic review and meta-analysis
Source: Sci Rep. 2022 Feb 8;12:2101. doi: 10.1038/s41598-022-05988-0 (PMC8825843; doi:10.1038/s41598-022-05988-0)
Supplement: Supplementary file 1 — Supplementary Information. [file 41598_2022_5988_MOESM1_ESM.pdf]

Title

Clinical outcomes, complications and fusion rates in endoscopic assisted intraforaminal lumbar interbody fusion (iLIF) versus minimally invasive transforaminal lumbar interbody fusion (MI-TLIF): systematic review and meta-analysis.

Authors

*José Miguel Sousa[1, 2]

https://orcid.org/0000-0002-1790-8213

Corresponding author. E-mail: josemiguelsousa.spine@gmail.com

Hugo Ribeiro[1]

https://orcid.org/0000-0002-5736-5778

João Luís Silva[1]

https://orcid.org/0000-0003-4042-1224

Paulo Nogueira[3]

https://orcid.org/0000-0001-8316-5035

José Guimarães Consciência[1, 2]

https://orcid.org/0000-0002-7633-0589

1. Centro Hospitalar Lisboa Ocidental, Orthopaedics Department, Lisbon, Portugal

Address

Estrada do Forte do Alto Duque, 1449-005 Lisboa, Portugal

2. NOVA Medical School - Universidade NOVA de Lisboa, Lisbon, Portugal

Address

Campo Mártires da Pátria, 130, 1169-056 Lisboa, Portugal

3. Instituto de Medicina Preventiva e Saúde Pública, Faculdade de Medicina, Universidade de Lisboa, Área Disciplinar Autónoma de Bioestatística (Laboratório de Biomatemática), Lisbon, Portugal

Address

Avenida Professor Egas Moniz, 1649-028 Lisboa, Portugal

PRISMA 2009 Checklist

Section/topic	#	Checklist item	Reported on page #
**TITLE**			
Title	1	Identify the report as a systematic review, meta-analysis, or both.	1
**ABSTRACT**			
Structured summary	2	Provide a structured summary including, as applicable: background; objectives; data sources; study eligibility criteria, participants, and interventions; study appraisal and synthesis methods; results; limitations; conclusions and implications of key findings; systematic review registration number.	2
**INTRODUCTION**			
Rationale	3	Describe the rationale for the review in the context of what is already known.	3
Objectives	4	Provide an explicit statement of questions being addressed with reference to participants, interventions, comparisons, outcomes, and study design (PICOS).	3, 4
**METHODS**			
Protocol and registration	5	Indicate if a review protocol exists, if and where it can be accessed (e.g., Web address), and, if available, provide registration information including registration number.	4
Eligibility criteria	6	Specify study characteristics (e.g., PICOS, length of follow-up) and report characteristics (e.g., years considered, language, publication status) used as criteria for eligibility, giving rationale.	4-5
Information sources	7	Describe all information sources (e.g., databases with dates of coverage, contact with study authors to identify additional studies) in the search and date last searched.	4
Search	8	Present full electronic search strategy for at least one database, including any limits used, such that it could be repeated.	4
Study selection	9	State the process for selecting studies (i.e., screening, eligibility, included in systematic review, and, if applicable, included in the meta-analysis).	4-5
Data collection process	10	Describe method of data extraction from reports (e.g., piloted forms, independently, in duplicate) and any processes for obtaining and confirming data from investigators.	5
Data items	11	List and define all variables for which data were sought (e.g., PICOS, funding sources) and any assumptions and simplifications made.	5
Risk of bias in individual studies	12	Describe methods used for assessing risk of bias of individual studies (including specification of whether this was done at the study or outcome level), and how this information is to be used in any data synthesis.	5-6
Summary measures	13	State the principal summary measures (e.g., risk ratio, difference in means).	6
Synthesis of results	14	Describe the methods of handling data and combining results of studies, if done, including measures of consistency (e.g., I^2) for each meta-analysis.	6

PRISMA 2009 Checklist

Section/topic	#	Checklist item	Reported on page #
Risk of bias across studies	15	Specify any assessment of risk of bias that may affect the cumulative evidence (e.g., publication bias, selective reporting within studies).	5, 6
Additional analyses	16	Describe methods of additional analyses (e.g., sensitivity or subgroup analyses, meta-regression), if done, indicating which were pre-specified.	6
**RESULTS**			
Study selection	17	Give numbers of studies screened, assessed for eligibility, and included in the review, with reasons for exclusions at each stage, ideally with a flow diagram.	7, Fig 1
Study characteristics	18	For each study, present characteristics for which data were extracted (e.g., study size, PICOS, follow-up period) and provide the citations.	7, Table 1
Risk of bias within studies	19	Present data on risk of bias of each study and, if available, any outcome level assessment (see item 12).	Appendix 1
Results of individual studies	20	For all outcomes considered (benefits or harms), present, for each study: (a) simple summary data for each intervention group (b) effect estimates and confidence intervals, ideally with a forest plot.	7, 8 Tables 2, 3, 5, 6
Synthesis of results	21	Present results of each meta-analysis done, including confidence intervals and measures of consistency. *Present the main results of the review. If meta-analyses are done, include for each, confidence intervals and measures of consistency*	7, 8, 9 Tables 4, 5, 7, 9
Risk of bias across studies	22	Present results of any assessment of risk of bias across studies (see Item 15).	6, 7
Additional analysis	23	Give results of additional analyses, if done (e.g., sensitivity or subgroup analyses, meta-regression [see Item 16]).	9
**DISCUSSION**			
Summary of evidence	24	Summarize the main findings including the strength of evidence for each main outcome; consider their relevance to key groups (e.g., healthcare providers, users, and policy makers).	10, 11
Limitations	25	Discuss limitations at study and outcome level (e.g., risk of bias), and at review-level (e.g., incomplete retrieval of identified research, reporting bias).	12
Conclusions	26	Provide a general interpretation of the results in the context of other evidence, and implications for future research.	13
**FUNDING**			
Funding	27	Describe sources of funding for the systematic review and other support (e.g., supply of data); role of funders for the systematic review.	Disclosures

From: Moher D, Liberati A, Tetzlaff J, Altman DG, The PRISMA Group (2009). Preferred Reporting Items for Systematic Reviews and Meta-Analyses: The PRISMA Statement. PLoS Med 6(7): e1000097. doi:10.1371/journal.pmed1000097

For more information, visit: www.prisma-statement.org.

Appendix Table 1 MINORS scores of included studies

	MINORS	1. A clearly stated aim	2. Inclusion of consecutive patients	3. Prospective collection of data	4. Endpoints appropriate to the aim of the study:	5. Unbiased assessment of the study endpoint	6. Follow-up period appropriate to the aim of the	7. Loss to follow up less than 5%	8. Prospective calculation of the study size	9. An adequate control group:	10. Contemporary groups	11. Baseline equivalence of groups	12. Adequate statistical analyses
Park et al.	13	2	2	2	2	2	2	1	0				
Rouben	13	2	2	2	2	1	2	2	0				
Lee et al	22	2	2	2	2	2	2	2	0	2	2	2	2
Lee	14	2	2	2	2	2	2	2	0				
Saetia	13	2	2	1	2	2	2	2	0				
Seng	22	2	2	2	2	2	2	2	0	2	2	2	2
Gu et al.	14	2	2	2	2	2	2	2	0				
Lee	24	2	2	2	2	2	2	2	2	2	2	2	2
Min	18	2	0	0	2	2	2	2	0	2	2	2	2
Shen et al.	19	2	0	1	2	2	2	2	0	2	2	2	2
Adogwa et al	20	2	2	2	0	2	2	2	0	2	2	2	2
Brodano et al.	21	2	2	1	2	2	2	2	0	2	2	2	2
Kuo et al.	22	2	2	2	2	2	2	2	0	2	2	2	2
Li et al	22	2	2	2	2	2	2	2	0	2	2	2	2
Yang et al.	24	2	2	2	2	2	2	2	2	2	2	2	2
Fan et al	22	2	2	2	2	2	2	2	0	2	2	2	2
Gao et al.	19	2	1	1	2	2	2	1	0	2	2	2	2
Kim et al	14	2	2	2	2	2	2	2	0				
Shen et al.	20	2	0	2	2	2	2	2	0	2	2	2	2

Lv et al	20	2	0	2	2	2	2	2	0	2	2	2	2
Razak et al	14	2	2	2	2	2	2	2	0				
Serban et al	19	1	1	2	1	2	2	2	0	2	2	2	2
Yang et al	14	2	2	2	2	2	2	2	0				
Yang et al	20	2	0	2	2	2	2	2	0	2	2	2	2
Zhang et al	19	2	0	1	2	2	2	2	0	2	2	2	2
Wu et al.	17	1	0	0	2	2	2	2	0	2	2	2	2
Wu et al.	11	2	2	2	0	2	2	1	0				
Zhao et al.	20	2	2	0	2	2	2	2	0	2	2	2	2
Goh et al.	20	2	2	2	0	2	2	2	0	2	2	2	2
Kolcun et al.	13	2	2	2	2	2	2	1	0				
Lin et al.	22	2	2	2	2	2	2	2	0	2	2	2	2
Mokawem et al.	22	2	2	2	2	2	2	2	0	2	2	2	2
Shen et al.	12	2	2	2	0	2	2	2	0				
Yang et al.	12	2	0	2	2	2	2	2	0				
Zhao et al	12	2	2	2	2	0	2	2	0				
Zhao et al.	19	2	0	1	2	2	2	2	0	2	2	2	2
Ao et al.	22	2	2	2	2	2	2	2	0	2	2	2	2
Chan et al.	22	2	2	2	2	2	2	2	0	2	2	2	2
El Mansy et al.	22	2	2	2	2	2	2	2	0	2	2	2	2
Jin et al.	10	2	2	0	2	0	2	2	0				
Kim et al.	21	2	2	2	2	1	2	2	0	2	2	2	2
Wang et al.	11	2	1	2	2	0	2	2	0				

Items were scored as 0 (not reported), 1 (reported but inadequate), or 2 (reported and adequate), with a maximum score of 16 for noncomparative studies and 24 for comparative studies.
MINORS, Methodological Index for Non-randomized Studies
